# Preparation of Carboxylato-Coordinated Titanium Alkoxides from Carboxylic Anhydrides: Alkoxido Group Transfer from Metal Atom to Carbonyl Group

**DOI:** 10.1002/ejic.201200296

**Published:** 2012-06-13

**Authors:** Matthias Czakler, Christine Artner, Ulrich Schubert

**Affiliations:** [a] Institute of Materials Chemistry, Vienna University of Technology Getreidemarkt 91060 Wien, Austria

**Keywords:** Titanium, Alkoxides, Carb­oxylic anhydrides, Carboxylate ligands, Hydrolysis

## Abstract

Reaction of titanium(IV) isopropoxide, Ti(O*i*Pr)_4_, with an equimolar amount of phthalic anhydride resulted in the transfer of an isopropoxido group from the metal atom to one carbonyl group of the anhydride and coordination of the thus formed monoester to the titanium atom. One monoester ligand in Ti_2_(O*i*Pr)_6_(μ_2_-OOC-C_6_H_4_-COO*i*Pr)(η^1^-OOC-C_6_H_4_-COO*i*Pr)(*i*PrOH) is bridging and the other is η^1^-coordinated. When the reaction is performed in the presence of 1 mol-equiv. of acetic acid, the oxido cluster Ti_6_(μ_3_-O)_6_(O*i*Pr)_6_(μ_2_-OOC-C_6_H_4_-COO*i*Pr)_6_ was instead obtained. The μ_3_-oxygen groups in the latter compound are due to esterification of acetic acid by the cleaved isopropyl alcohol.

## Introduction

Carboxylic acids are frequently used to moderate the reactivity of metal alkoxides, M(OR)*_n_*, for sol-gel processing. This is not a straightforward reaction, however, because oxido clusters of the type M*_a_*O*_b_*(OH/OR)*_c_*(OOCR′)*_d_* are often obtained instead of the substitution products M(OR)_*n*–*x*_(OOCR′)*_x_*.^1^,^2^ The case of Ti(OR)_4_ has been particularly well investigated. Spectroscopic analysis of the reaction of Ti(OBu)_4_ with an equimolar quantity of acetic acid suggested that [Ti(OR)_3_(OOCR′)]*_n_* (*n* = 2 or 3) was formed and that the acetate ligands were bridging.^3^ A corresponding structure was found for crystalline Ti_2_(OCH_2_CMe_3_)_6_(OOC-CMe_3_)_2_, with bulky alkoxido and carboxylato ligands.^4^The compound Ti_2_(O*i*Pr)_6_(μ_2_-OOC-CMe_3_)(η^1^-OOC-CMe_3_)(*i*PrOH) possibly represents an intermediate state in the coordination of Ti(OR)_4_ by the second carboxylato ligand. One carboxylato ligand is only monodentate, and the vacant coordination site at the other titanium atom is occupied by an isopropyl alcohol molecule hydrogen-bonded to the dangling oxygen atom of the monodentate carboxylato ligand.^4^ In most reactions of Ti(OR)_4_ with carboxylic acids, however, carboxylato-coordinated oxido/alkoxido clusters Ti*_a_*O*_b_*(OR)*_c_*(OOCR′)*_d_* have been obtained.^2^

The most probable sequence of reactions leading to the formation of such clusters is that one or more alkoxido ligands are replaced by carboxylato ligands in the first step (see above), followed by esterification of the liberated alcohol. The thus produced water could be the source of oxido or hydroxido ligands in the clusters. This possibility is also supported by a few cases, where compounds with coordinated water molecules were isolated from such reactions, such as Zr_6_O_4_(OH)_4_(isobutyrate)_12_(H_2_O)^5^ or a series of hydrated yttrium carboxylates.^6^ The carboxylic acids employed in the reactions with metal alkoxides thus have a dual role: as a source for the bidentate ligands to cap the core of the formed clusters and to provide water through esterification reactions.

In the work reported in this article, we attempted to decouple these two functions. To this end, we treated a carboxylic anhydride with Ti(O*i*Pr)_4_. We chose the anhydride of a dicarboxylic acid (phthalic anhydride), because the groups formed during the reaction are linked with each other and thus easier to identify. The results reported in this article provide new insight into reactions of metal alkoxides with carboxylic acids.

## Results and Discussion

**Figure 1:**
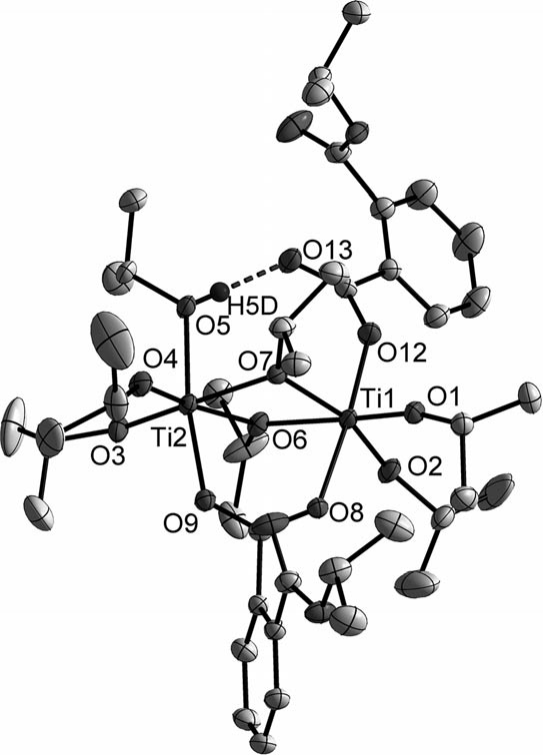
Molecular structure of Ti_2_(O*i*Pr)_6_(μ_2_-OOC-C_6_H_4_-COO*i*Pr)(η^1^-OOC-C_6_H_4_-COO*i*Pr)(*i*PrOH) (**1**). Selected distances [pm] and angles [°]: Ti(1)–Ti(2) 322.74(6), Ti(1)–O(1) 177.82(13), Ti(1)–O(2) 181.07(13), Ti(1)–O(6) 205.37(12), Ti(1)–O(7) 204.92(12), Ti(1)–O(8) 210.41(11), Ti(1)–O(12) 198.09(12), Ti(2)–O(3) 181.92(12), Ti(2)–O(4) 177.34(12), Ti(2)–O(5) 207.20(12), Ti(2)–O(6) 202.85(11), Ti(2)–O(7) 202.86(12), Ti(2)–O(9) 206.17(11); Ti(1)–O(6)–Ti(2) 104.48(5), Ti(1)–O(7)–Ti(2) 104.64(5), O(7)–Ti(1)–O(6) 72.82(5), O(6)–Ti(1)–O(8) 85.80(5), O(7)–Ti(1)–O(8) 84.57(5), O(1)–Ti(1)–O(6) 167.30(5), O(2)–Ti(1)–O(7) 163.25(5), O(12)–Ti(1)–O(8) 175.49(5), O(4)–Ti(2)–O(6) 168.56(5), O(3)–Ti(2)–O(7) 166.26(5), O(6)–Ti(2)–O(7) 73.78(5), O(9)–Ti(2)–O(5) 171.52(5).

Reaction of Ti(O*i*Pr)_4_ with an equimolar amount of phthalic anhydride in isopropyl alcohol resulted in the quantitative formation of crystalline Ti_2_(O*i*Pr)_6_(μ_2_-OOC-C_6_H_4_-COO*i*Pr)(η^1^-OOC-C_6_H_4_-COO*i*Pr)(*i*PrOH) (**1**) [Equation (1)]. The molecular structure of **1** is analogous to that of Ti_2_(O*i*Pr)_6_(μ_2_-OOC-CMe_3_)(η^1^-OOC-CMe_3_)(*i*PrOH) mentioned before.^4^ The two titanium atoms in the asymmetric dimer are bridged by two O*i*Pr ligands and one phthalate monoester ligand (Figure 1). The octahedral coordination of Ti(1) is completed by coordination of an η^1^-phthalate monoester and two terminal O*i*Pr ligands, and that of Ti(2) by three terminal O*i*Pr ligands.


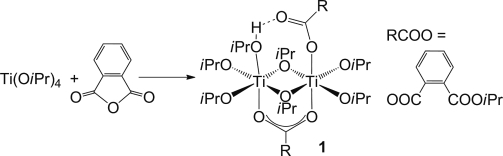
(1)

Because of the bridging carboxylato ligand, the [TiO_6_] coordination octahedra are slightly tilted towards this ligand. The carbonyl oxygen O(13) of the η^1^-phthalic ester and that of the adjacent O*i*Pr ligand O(5) at the neighboring titanium atom are clearly connected through a hydrogen bond [O(5)**···**O(13) 260.3(2) pm]. The hydrogen atom was located in difference Fourier maps, and was close to O(5) of the O*i*Pr ligand [O(5)–H(5D) 81(3) pm] with an O(5)–H(5D)–O(13) angle of 175(3)°, corresponding to a coordinated *i*PrOH molecule. This can also be concluded from the Ti(2)–O(5) distance [207.20(12) pm], which is much longer than that of the Ti–O distances of the other terminal O*i*Pr ligands (177.8–181.9 pm). Furthermore, the C(33)–O(13) distance is 124.2(2) pm, which is only slightly longer than that of the ester CO group in the bridging ligand [C(29)–(O11) 120.4(2) pm]. The stabilization of an η^1^-carboxylato ligand by means of a hydrogen bond to the oxygen atom of an adjacent ligand was also observed in Zr_6_O_4_(OH)_4_(isobutyrate)_12_(HX) (HX = H_2_O or BuOH).^5^ Solution NMR spectroscopic data are in line with the solid-state structure, but a clear assignment of individual signals (especially in the phenyl and O*i*Pr areas) is not possible due to the different groups with similar chemical shifts.

Although the general structure type represented by **1** has been observed before (see above), the formation of **1** is remarkable. Substitution of metal alkoxides is usually performed by reaction with protic compounds (HY) during which the proton of HY is transferred to an OR group with subsequent elimination of ROH (Scheme 1; X = H, for Y = OOCR′). In a similar manner, an alkoxido group could be transferred to the R′C(O) moiety of the anhydride with concomitant formation of an ester (Scheme 1; X = R′CO). The resulting carboxylato ligand is coordinated to the titanium atom. If a cyclic anhydride is used, as in the case of phthalic anhydride, the ester and carboxylate groups remain of course attached to each other.

**Figure:**
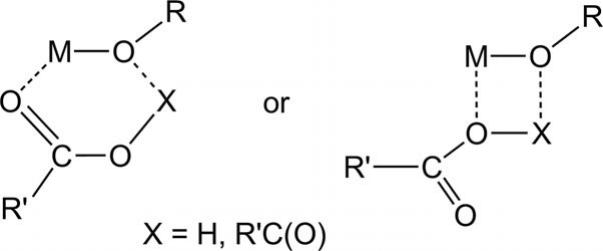
Scheme 1.

No protic compounds are involved in the reaction with phthalic anhydride. No water or hydroxido groups can therefore be formed, and hence no oxido/hydroxido clusters. To gain further insight into the role of carboxylic acids in reactions with metal alkoxides, we modified the above reaction in a way that an equimolar amount of acetic acid was added to the isopropyl alcohol solution of Ti(O*i*Pr)_4_ and phthalic anhydride. This allowed a competition between a carboxylic acid and a carboxylic anhydride as potential sources for carboxylato ligands.

After a long reaction period, crystals of the centrosymmetric cluster Ti_6_O_6_(O*i*Pr)_6_(OOC-C_6_H_4_-COO*i*Pr)_6_ (**2**) were isolated [Equation (2)]. The IR spectrum of the supernatant solution showed a broad peak at 1723 cm^–1^ indicating ester formation. For comparison: the ν_CO_ band of isopropyl acetate is at 1735 cm^–1^ and that of acetic acid at 1710 cm^–1^. The cluster core of **2** can be described as a Ti_6_ octahedron in which six of the eight triangular faces are capped by μ_3_-oxygen atoms or as a slightly distorted hexagonal prism with alternating titanium and oxygen atoms. The six phthalate isopropyl ester ligands bridge the six four-membered Ti_2_O_2_ rings of the hexagonal prism. Each titanium atom is octahedrally coordinated by two phthalate ester groups, one terminal O*i*Pr ligand and three μ_3_-oxygen atoms. The terminal OR ligands are oriented perpendicular to the slightly puckered Ti_3_O_3_ rings. This structural motif was already found in other Ti_6_O_6_ clusters obtained by reaction of titanium alkoxides with various carboxylic acids^7^ or oximes.^8^ The bond lengths and angles observed in **2** are in the same range as in the reference compounds.


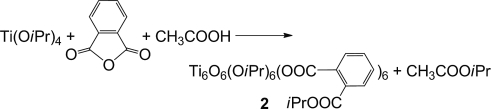
(2)

**Figure 2:**
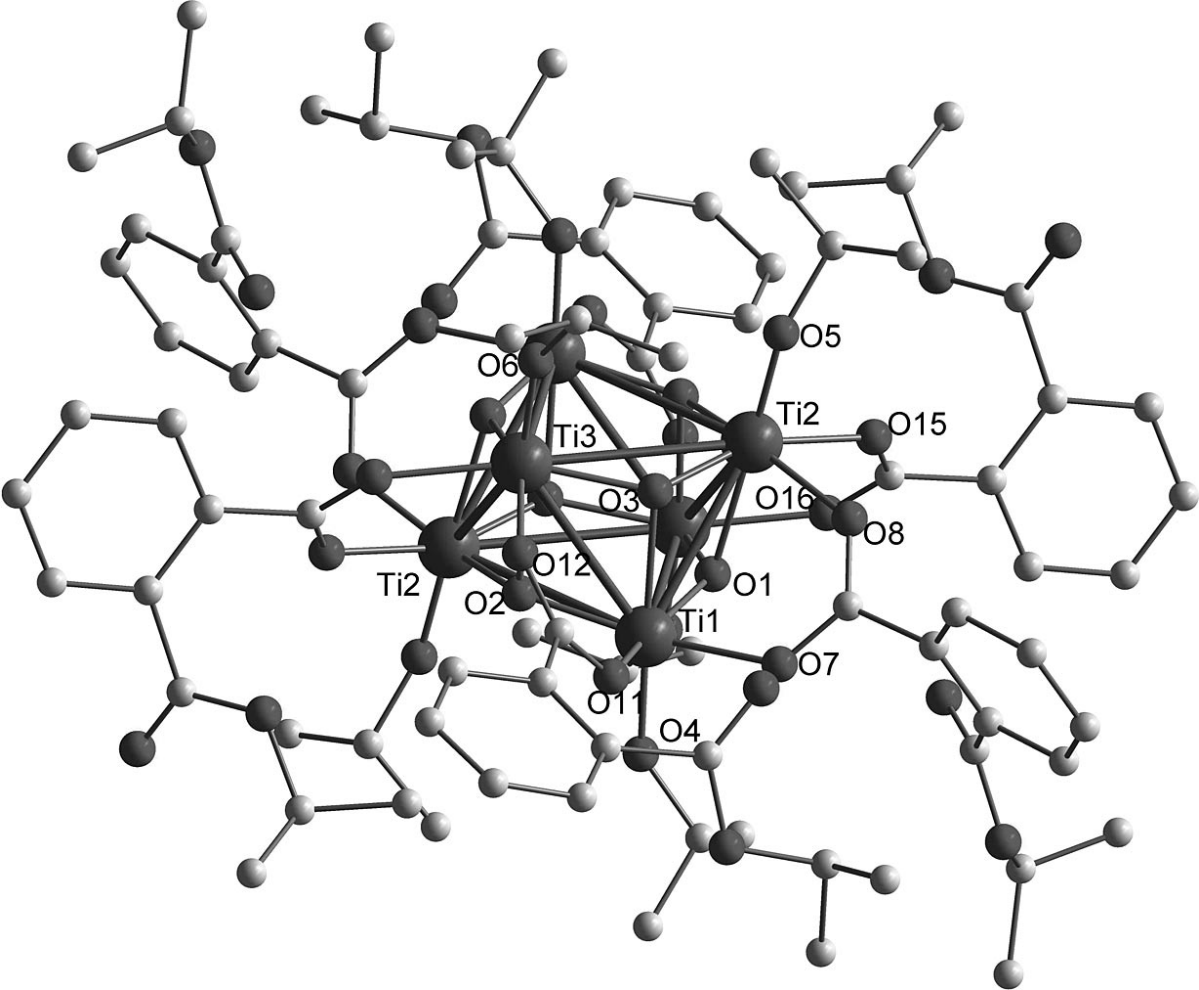
Molecular structure of Ti_6_O_6_(O*i*Pr)_6_(OOC-C_6_H_4_-COO*i*Pr)_6_ (**2**). Selected distances [pm] and angles [°]: Ti(1)–O(1) 190.3(4), Ti(1)–O(2) 187.0(4), Ti(1)–O(3) 213.7(4), Ti(1)–O(4) 176.0(4), Ti(1)–O(7) 205.6(5), Ti(1)–O(11) 206.4(4), Ti(2)–O(1) 215.5(4), Ti(2)–O(2) 192.1(4), Ti(2)–O(3) 187.3(4), Ti(2)–O(5) 174.7(4), Ti(2)–O(8) 205.0(5), Ti(2)–O(15) 207.7(4), Ti(3)–O(1) 188.5(4), Ti(3)–O(2) 216.8(4), Ti(3)–O(3) 192.5(4), Ti(3)–O(6) 176.6(4), Ti(3)–O(12) 206.3(5), Ti(3)–O(16) 208.2(4); O(1)–Ti(1)–O(2) 103.0(2), O(1)–Ti(1)–O(3) 77.5(2), O(1)–Ti(1)–O(4) 99.7(2), O(1)–Ti(1)–O(7) 86.0(2), O(1)–Ti(1)–O(11) 158.6(2), O(1)–Ti(2)–O(2) 78.0(2), O(1)–Ti(2)–O(3) 77.7(2), O(1)–Ti(2)–O(5) 177.8(2), O(1)–Ti(2)–O(8) 88.5(2). O(1)–Ti(2)–O(15) 88.1(2), O(1)–Ti(3)–O(2) 78.4(2), O(1)–Ti(3)–O(3) 101.4(2), O(1)–Ti(3)–O(6) 105.3(2), O(1)–Ti(3)–O(12) 157.9(2), O(1)–Ti(3)–O(16) 86.5(2).

Compound **2** only contains phthalate monoester ligands but no acetato ligand (Figure 2). This is remarkable, because two potential sources for carboxylato ligands were present in the reaction mixture (in a 1:1 ratio). It is rather obvious that the phthalate monoester ligands were formed by the same reaction as in **1**. On the other hand, the μ_3_-oxygen atoms in **2** must be due to esterification of acetic acid. Since formation of **2** was much slower than that of **1**, it can be assumed that reaction of the anhydride (giving the phthalate monoester ligands) is faster than that of acetic acid (resulting in partial hydrolysis). Although we could not identify other species, which may be present in the reaction solution, it appears that the role of acetic acid was to provide the water for *hydrolysis* of part of the alkoxido groups (through formation of isopropyl acetate), while phthalic anhydride served to *replace* part of the alkoxido groups.

## Conclusions

The results reported in this paper strongly support the notion that the first step of the reaction of Ti(OR)_4_ with carboxylic acids is the formation of carboxylato-coordinated titanium alkoxides Ti(OR)_*n*–*x*_(OOCR′)*_x_* (*x* = 1 or 2). In the case studied here, i.e. the reaction of a carboxylic anhydride, the carboxylato ligand was formed by a route that excludes the formation of water or OH species and thus the formation of oxido/hydroxido clusters. The carboxylato ligand, which eventually was coordinated to the titanium atom, was generated by OR group transfer from the metal atom to the other CO group of the anhydride. Formation of **1** was a surprisingly fast and quantitative process.

When the system was modified in a way that slow internal water production was possible (with otherwise the same reaction conditions), the oxido/alkoxido cluster **2** was obtained instead. The added acetic acid is the only possible source of the oxido groups. If one assumes that formation of **1** is the first step in the formation of **2** (because formation of **1** is very fast), two O*i*Pr ligands must be subsequently be replaced by one oxido groups, while the Ti/OOCR ratio is retained. The formal equation 2 *i*PrO^–^ + CH_3_COOH → CH_3_COO*i*Pr + *i*PrOH + O^2–^ shows that 1 mol-equiv. of acetic acid per titanium is sufficient to explain the outcome of the overall reaction.

## Experimental Section

**General:** All compounds were handled under argon by using standard Schlenk techniques. Isopropyl alcohol was dried by distilling twice from sodium, acetic acid was freshly distilled from P_2_O_5_ prior to use, Ti(O*i*Pr)_4_ and phthalic anhydride were used as received from Aldrich.

**Ti_2_(O*i*Pr)_6_(μ_2_-OOC-C_6_H_4_-COO*i*Pr)(η^1^-OOC-C_6_H_4_-COO*i*Pr)(*i*PrOH) (1):** Ti(O*i*Pr)_4_ (1.08 g, 3.8 mmol) was added to a suspension of phthalic anhydride (560 mg, 3.8 mmol) in isopropyl alcohol (291 μL, 3.8 mmol). The mixture was heated until a clear solution was obtained. Crystals were obtained at room temperature within 24 h. Yield 1.86 g (100 %). ^1^H NMR (CD_2_Cl_2_, 300 MHz): *δ* = 1.29 (d, *J* = 6.1 Hz, 42 H, CH_3_), 1.39 (d, *J* = 6.0 Hz, 12 H, CH_3_, isopropyl ester), 4.60–5.20 (7 H, C*H*, O*i*Pr), 5.25 (m, 2 H, C*H*, isopropyl ester), 7.3–7.7 (m, 6 H, C*H*, Ph), 8.12 (2 H, C*H*-C-COOTi) ppm. ^13^C NMR (CD_2_Cl_2_, 75 MHz): *δ* = 21.4 (*C*H_3_, ester), 24.71 (*C*H_3_, *i*PrOH, H-bond), 25.55 (*C*H_3_, O*i*Pr), 68.4 (*C*H, ester, H-bonded), 69.3 (*C*H, ester), 76.2–78.2 (*C*H, O*i*Pr, terminal), 79–81 (*C*H, O*i*Pr, bridging), 127–136 (*C*H, aryl), 166.6 (*C*OO, ester), 167.8 (*C*OO, ester, H-bonded), 182.5 (*C*OO-Ti) ppm. IR: 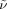 = 2972 (w, C–H), 1723 (m, C=O, ester), 1550 (m, C=O, acetate), 1493 (w), 1403 (m), 1290 (m), 1109 (s), 1076 (m), 1011 (m), 851 (w), 822 (w) cm^–1^.

**Ti_6_O_6_(O*i*Pr)_6_(OOC-C_6_H_4_-COO*i*Pr)_6_ (2):** Ti(O*i*Pr)_4_ (1.2 g, 4.25 mmol) was added to a suspension of phthalic anhydride (630 mg, 4.25 mmol) in a mixture of acetic acid (255 mg, 4.25 mmol) and isopropyl alcohol (1 mL, 17 mmol). The suspension was heated until a clear solution was obtained. Colorless crystals were obtained at room temperature after 10 weeks. Yield 600 mg (43 %). ^1^H NMR (CD_2_Cl_2_, 250 MHz): *δ* = 0.8–1.7 (72 H, C*H*_3_), 4.5–5.3 (12 H, C*H*), 7.1–8.1 (24 H, aryl) ppm. ^13^C NMR (CD_2_Cl_2_, 63 MHz): *δ* = 21–25 (*C*H_3_, O*i*Pr), 68.5–69.5 (*C*H, ester), 79–80 (*C*H, O*i*Pr), 128–135 (*C*, aryl), 166–168 (*C*OO*i*Pr, ester) 174–180 (*C*OO, acetate) ppm. IR: 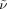 = 2972 (w, C–H), 1726 (m, C=O, ester), 1555 (m, C=O, acetate), 1400 (s), 1275 (m), 1108 (m), 1075 (m), 1008 (m), 950 (m), 840 (m) cm^–1^.

**X-ray Structural Analyses:** All measurements were performed at 100 K by using Mo-*K*_α_ (*λ* = 0.71073 Å) radiation. Data was collected with a Bruker AXS SMART APEX II four-circle diffractometer with κ-geometry. Data were collected with ϕ- and ω-scans and 0.5° frame width. The data were corrected for polarization and Lorentz effects, and an empirical absorption correction (SADABS)^9^ was employed. The cell dimensions were refined with all unique reflections. SAINT PLUS software^10^ was used to integrate the frames. Symmetry was then checked with the program PLATON.^11^ Details of the X-ray investigations are given in Table 1. The structures were solved by the Patterson method (SHELXS-97).^12^ Refinement was performed by the full-matrixleast-squares method based on *F*^2^ (SHELXL-97)^13^ with anisotropic thermal parameters for all non-hydrogen atoms. Hydrogen atoms were inserted in calculated positions and refined riding with the corresponding atom, those bonded to oxygen atoms were identified in the electron density map. The carbon atoms of almost all O*i*Pr ligands of **1** were disordered, especially that of the non-bridging O*i*Pr. Their positions were refined with two sites, with about 50 % occupancy each. CCDC-862908 (for **1**) and -CCDC-862909 (for **2**) contain the supplementary crystallographic data for this paper. These data can be obtained free of charge from The Cambridge Crystallographic Data Centre via www.ccdc.cam.ac.uk/data_request/cif.

**Table 1:** Crystallographic data for **1** and **2**

	1	2
Empirical formula	C_43_H_71_O_15_Ti_2_	C_84_H_108_O_36_Ti_6_
*M*_r_	923.8	1981.1
Crystal system	triclinic	triclinic
Space group	*P* 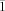	*P* 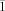
*a* [pm]	1148.9(2)	1290.95(7)
*b* [pm]	1177.70(18)	1368.20(8)
*c* [pm]	1944.0(3)	1458.24(7)
*α* [°]	103.090(8)	85.570(4)
*β* [°]	97.570(9)	66.340(4)
*γ* [°]	99.780(8)	78.120(4)
*V* [pm^3^ × 10^6^]	2484.7(7)	2308.6(2)
*Z*	2	1
*D_x_* [Mg m^–3^]	1.236	1.425
*μ* [mm^–1^]	0.382	0.579
Crystal size [mm]	0.4 × 0.32 × 0.3	0.2 × 0.18 × 0.14
No. measured, independent, observed refl. [*I* > 2σ (*I*)]	72908, 14419, 11404	11300, 6535, 3965
*R*_*i*n*t*_	0.0356	0.0476
*θ*_max_ [°]	30.01	23.25
*R* [*F*^2^ > 2σ(*F*)], *wR* (*F*^2^), *S*	0.0439, 0.1206, 1.028	0.0670, 0.2028, 1.027
No. reflections/parameters	14419/635	6535/580
Weighting scheme	*w* = 1/[σ^2^(*F*_o_^2^) + (0.0529*P*)^2^ + 1.8451*P*][a]	*w* = 1/[σ^2^(*F*_o_^2^) + (0.1108*P*)^2^ + 1.4297*P*][a]
*δρ*_max_, *δρ*_min_ [e Å^–3^]	1.312, –0.693	0.946, –0.602

[a] *P* = (*F*_o_^2^ + 2*F*_c_^2^)/3.
